# Integrated Analysis of mRNA- and miRNA-Seq in the Ovary of Rare Minnow *Gobiocypris rarus* in Response to 17α-Methyltestosterone

**DOI:** 10.3389/fgene.2021.695699

**Published:** 2021-08-05

**Authors:** Shaozhen Liu, Qiong Yang, Yue Chen, Qing Liu, Weiwei Wang, Jing Song, Yao Zheng, Wenzhong Liu

**Affiliations:** ^1^College of Animal Science, Shanxi Agriculture University, Jinzhong, China; ^2^Freshwater Fisheries Research Center, Chinese Academy of Fishery Sciences, Wuxi, China

**Keywords:** 17α-methyltestosterone, *Gobiocypris rarus*, RNA-seq, miRNA-seq, ovary

## Abstract

17α-Methyltestosterone (MT) is a synthetic androgen. The objective of this study was to explore the effects of exogenous MT on the growth and gonadal development of female rare minnow *Gobiocypris rarus.* Female *G. rarus* groups were exposed to 25–100 ng/L of MT for 7 days. After exposure for 7 days, the total weight and body length were significantly decreased in the 50-ng/L MT groups. The major oocytes in the ovaries of the control group were vitellogenic oocytes (Voc) and cortical alveolus stage oocytes (Coc). In the MT exposure groups, some fish had mature ovaries with a relatively lower proportion of mature oocytes, and the diameter of the perinucleolar oocytes (Poc) was decreased compared with those of the control group. Ovarian VTG, FSH, LH, 11-KT, E2, and T were significantly increased after exposure to 50 ng/L of MT for 7 days. Unigenes (73,449), 24 known mature microRNAs (miRNAs), and 897 novel miRNAs in the gonads of *G. rarus* were found using high-throughput sequencing. Six mature miRNAs (miR-19, miR-183, miR-203, miR-204, miR-205, and miR-96) as well as six differentially expressed genes (*fabp3*, *mfap4*, *abca1*, *foxo3*, *tgfb1*, and *zfp36l1*) that may be associated with ovarian development and innate immune response were assayed using qPCR. Furthermore, the miR-183 cluster and miR-203 were differentially expressed in MT-exposed ovaries of the different *G. rarus* groups. This study provides some information about the role of miRNA–mRNA pairs in the regulation of ovarian development and innate immune system, which will facilitate future studies of the miRNA–RNA-associated regulation of teleost reproduction.

## Introduction

17α-Methyltestosterone (MT) is a synthetic organic compound, which is a typical endocrine disruptor widely available in the environment. MT is commonly found in sewage from paper mill and domestic and livestock manure, especially in aquatic reproduction; approximately 1.33 ng/L of MT was detected in the sewage discharge of chemical plants, while 4.1–7.0 ng/L MT was detected in Beijing wastewater samples. MT can inhibit the activity of steroidogenic enzymes, especially aromatase (Kortner and Arukwe, 2007), and cause alterations in sex steroid hormone levels in the body (Lyu et al., 2019; Meng et al., 2019). MT exposure can, thus, lead to sex reversal in many kinds of fish, such as zebrafish (Lee et al., 2017) and orange-spotted grouper (Wang et al., 2018; Huang et al., 2019; Lyu et al., 2019). When 7-month-old Pengze *Carassius auratus* were exposed to MT (50 and 100 μg/L) in semistill water, they exhibited ovaries that were degenerated and atretic in both groups. Moreover, MT impaired the gonad development of *C. auratus* (Zheng et al., 2016) and the rare minnow species *Gobiocypris rarus* (25–100 ng/L) (Gao et al., 2014, 2015; Liu S. et al., 2014; Liu Y. et al., 2014; Liang and Zha, 2016). MT (302.5 ng/L) disturbed the gene expression of the hypothalamus–pituitary–gonadal axis in mummichog (*Fundulus heteroclitus*) (Rutherford et al., 2019). However, most studies, so far, have focused on gene expression without referring to the underlying microRNA (miRNA) regulation.

miRNAs are short (21–23 nucleotides), single-stranded, non-coding RNAs that form complimentary basepairs with the 3′ untranslated region of target mRNAs within the RNA-induced silencing complex (RISC) and block translation and/or stimulate mRNA transcript degradation (Kelly et al., 2013; Stavast and Erkeland, 2019). The total set of transcripts (mRNA and non-coding RNA) involved in the transcriptome is transcribed at a specific organization during a particular developmental stage (Mardis, 2008). In an organism, a single miRNA may control the expression of several genes, or multiple miRNAs work simultaneously to control the expression of a single gene (Bartel, 2004). Previous studies showed that miRNA may be an inducible factor to increase the complexity of organism with their roles in regulating gene expression (Heimberg et al., 2008). Recent studies have confirmed that miRNAs play an important role in regulating the development of fish embryos and hypoxia responses in the liver of darkbarbel catfish (Zhang et al., 2016), sex determination and differentiation in the gonad tissue of dark sleeper (Zhao et al., 2017), and fundamental cellular processes in the gut and liver of zebrafish (Renaud et al., 2018). In addition, miRNAs negatively regulate the function of genes associated with reproduction. For example, Bizuayehu et al. (2019) found that five biased miRNAs, ssa-let-7a, ssa-miR-10a, ssa-miR-20a, ssa-miR-130a, and ssa-miR-202, were related to the egg quality of Atlantic salmon (*Salmo salar*), while Fischer et al. (2019) found that FoxH1 repressed miR-430 in gonad development of zebrafish. Moreover, miRNAs dre-miR-143, dre-miR-101a, dre-miR-202-5p, dre-let-7c-5p, and dre-miR-181a-5p are related to gonad development of *Trachinotus ovatus* (He et al., 2019); ccr-miR-24, ccr-miR-146a, ccr-miR-192, ccr-miR-21, ccr-miR-143, and ccrmiR-454b regulate the gonad development of common carp (*Cyprinus carpio*) exposed to atrazine (Wang F. et al., 2019); and miRNA-26a/cyp19a1a regulates feedback loop in the protogynous hermaphroditic fish, *Epinephelus coioides* (Yu et al., 2019). Some recent studies have also found negative regulators (*let-7a/b/d*) of the ovary development process, in blunt snout bream (Lan et al., 2019), as well as those involved in steroid hormone synthesis related pathways, through miRNA–mRNA analysis in Japanese flounder (Zhang et al., 2019). Atrazine can upregulate aromatase expression through miRNAs, which supports the hypothesis that atrazine has endocrine-disrupting activity by altering the gene expression profile of the HPG axis through its corresponding miRNAs (Wang G. et al., 2019).

Some proteins, such as β-catenin in freshwater mussel *Hyriopsis cumingii* (Wang F. et al., 2019) and vitellogenin (VTG) in *Nothobranchius guentheri* (Liu et al., 2017), may participate in a variety of physiological activities like immune regulation and sex determination. BPA can increase the mRNA stability of β*-catenin* via suppressing the expression of miR-214-3p, which can directly target the 3′UTR of β*-catenin* mRNA (Zeng, 2020). Liu et al. (2021) found that the expression of *vtg* was regulated directly by miR-34, and the expression level of *vtg* in the agomiR-9c/-263a group was significantly decreased, while that in the antagomiR-9c/263a group, it was significantly increased. Such results indicate that miR-9c and miR-263a could regulate *vtg* indirectly by inhibiting the expression of *cyclins* and *CDKs*, thus, affecting the development of the ovary. Meanwhile, the changes in *vtg* expression are more intuitive to manifest that these genes can affect ovarian development through the regulation of miRNAs.

In this study, we tested the sex hormone (E2, T, FSH, LH, and 11-KT) and VTG of *G. rarus* exposed to MT (25, 50, and 100 ng/L) for 7 days. At the same time, we monitored whether there was a morphological change in the ovaries in rare minnow related to different concentrations of MT, thereby, investigating the differentiating effects of different concentrations of MT on ovarian development and follicle maturation in *G. rarus*. In this study, we aim to explore the negative regulatory effect of miRNA on mRNA expression after MT administration in order to find miRNA–target gene pairs. In addition, we will further study miRNA–mRNA interaction networks, which may help explore the underlying mechanisms of the reproduction and immune system of *G. rarus*.

## Materials and Methods

### Experimental Animals

All experiments for animals were approved by the Institutional Animal Care and Use Committee of Shanxi Agriculture University, and the IACUC No. is SXAU-EAW-2018G.R.0201. As MT is insoluble in water and only soluble in organic solvents, the MT stock solution was prepared in anhydrous ethanol. The experimental group of *G. rarus* from the same family was selected through artificial fertilization, and the females were segregated after sexual maturity according to distance between the hind fin and the tail fin because such distance of female fishes is longer than that of the male. In cases when it was difficult to distinguish sex by using external appearance, the ovaries of fishes were observed after the exposure experiment. Before the exposure experiment, 6-month-old female *G. rarus* were domesticated for 1 week after grouping. Then they were treated with different concentrations of MT (25, 50, and 100 ng/L named as MT25-F, MT50-F, and MT100-F, respectively, test groups) or with 0.0001% anhydrous ethanol (solvent control group) for 7 days. The concentration and exposure durations were determined according to our reports (Gao et al., 2014, 2015; Liu S. et al., 2014; Liu Y. et al., 2014) and the studies of Liang and Zha (2016) and Rutherford et al. (2019). All experiments were performed in triplicate for the three treatment and one control groups. A total of 12 aquariums were used, with a volume of 80 L each. There were 25–30 *G. rarus* females in each aquarium, ensuring the ratio of 1 g of fish for every 1 L of water. They were fed regularly and in fixed quantities every day. The method of semistatic water exposure was utilized to change half of the water in the aquarium, while sucking the sewage (residual bait and feces) and simultaneously adding the same amount of water along with the corresponding amount of MT solution, to ensure that the MT concentration in the aquarium remained unchanged.

### Measurement and Sampling of Biological Indicators

Three fish were selected from each aquarium and anesthetized. Biological indicators including the total length, body length, and body weight of all fish in the treatment and the control groups were measured (*n* = 6). The ovaries of six fish in each group (*n* = 6, a total of 18 fish from three repeated experiments) were halved and fixed with Bouin’s solution for ovarian morphological analysis of *G. rarus*. The ovaries were fixed for 24 h, following which, they were dehydrated with alcohol and treated with xylene to make the tissue transparent. The ovarian tissues were embedded in wax blocks. Continuous wax strips of 6 μm were prepared from these blocks using Leica M2245 (Germany, Leica Biosystems). H&E staining was performed, and the images were observed and photographed under an RCH1-NK50I light microscope. We used Image J v1.53a (Publisher Wayne Rasband) to count the number of cells in the H-E slices.

### ELISA

According to the methods reported by Lange et al. (2008), the whole body trunk (*n* = 3 per group) was taken from the severed tails via a capillary tube and quickly transferred to a heparinized centrifuge tube, and then the protease inhibitor (2 trypsin inhibitor units/ml) was added. Centrifugation was carried out at 21,380 × *g* for 15 min at −20°C. The supernatant was carefully pipetted out and stored at −80°C, for the determination of VTG, follicle-stimulating hormone (FSH), luteinizing hormone (LH), 11-ketotestosterone (11-KT), 17β-estradiol (E2), and testosterone (T) levels. These were determined using an ELISA kit (Nanjing Jiancheng Biotechnology Co., Ltd.). According to the protocols provided by the manufacturer, and for the specific steps, please refer to the instructions of such ELISA kits from Nanjing Jiancheng Biotechnology Co., Ltd. All samples and standards were run in triplicates (both the CV of inter-assay and intra-assay were less than 10%). When the standard curve of VTG, FSH, 11-KT, E2, LH, and T was *R*^2^ ≥ 0.99, it indicated that the experimental results are reliable.

### Samples for the Sequencing

We used trizol one-step method to extract the total RNA of one ovary of each fish, and then we mixed three fish RNA in the same aquarium to prepare a mixed sample, each group of three repeated tests to prepare three mixed samples, treatment group, and control group for a total of 12 samples (*n* = 12 groups in total with *n* = 3 per group) for mRNA (*n* = 3 per group with a total of nine individuals) and miRNA (*n* = 3 per group with a total of nine individuals) sequencing (Illumina HiSeqTM 2500, Guangzhou Gene *Denovo* Biotechnology Co.).

### RNA- and miRNA-Seq

Methods and protocols for total RNA extraction, RNA quantity assessment, library construction, and RNA sequencing were followed as provided in the previous studies (Liu S. et al., 2014; Gao et al., 2015; Renaud et al., 2018). The gene abundances were calculated and normalized to reads per kb per million (RPKM) using the DESeq2 and EBSeq software. “Up_diff” or “down_diff” were classified according to the RPKM values, whereas for miRNA-seq, all of the clean tags were mapped to the reference transcriptome (GenBank Release 209.0, Rfam database 11.0, miRBase database 21.0 for known miRNAs) to identify and remove rRNA, scRNA, snoRNA, snRNA, and tRNA from the miRNAs (Friedländer et al., 2012). The novel miRNA candidates were identified via the Mireap_v0.2 software, and the expression levels of both known and novel miRNAs were calculated and normalized to transcripts per million (TPM). Principal component analysis (PCA) was performed using R package models. MiRNA differential expression based on normalized deep sequencing counts was analyzed by *t*-test. Comparisons between control and MT-exposed groups were made to identify significant DEMs [| log_2_(fold change)| > 1 and *p* ≦ 0.05]. Data normalization followed the procedures as described in a previous study (Cer et al., 2014).

### Differentially Expressed Genes and DE miRNAs

We identified DEGs and DEMs with a fold change ≥ 2 and a false discovery rate (FDR) ≤ 0.05 among the comparisons across samples or groups. These common target genes from RNA- and miRNA-seq data were considered for further analysis. Gene Ontology (GO) enrichment and pathway-based analysis were simultaneously provided. RNAhybrid (v2.1.2) + svm_light (v6.01), Miranda (v3.3a), and TargetScan (Version 7.0, Agarwal et al., 2015) were used to predict the target gene–miRNA pairs.

Short time-series expression miner (STEM) was used to finally reveal the expression tendency of DEGs, and weighted gene co-expression network analysis (WGCNA v1.47, Filteau et al., 2013) was adopted to find the co-expression networks that were constructed. This study analyzed and identified the biological function of each miRNA–mRNA pair with a negative correlation.

### qPCR to Verify the Selected miRNAs

According to the results of the bioinformatics analysis, miR-19, miR-183, miR-203, miR-204, miR-205, miR-96, and their target genes in the immune process/steroidogenesis pathway were selected for qPCR validation (*n* = 3 per group). The cDNA was obtained by reverse transcription of total ovarian RNA, and the reverse transcription primers with a stem ring structure and their corresponding PCR primers were designed to detect the miRNA. The miRNA results were compared with the results of qPCR to determine the consistency of the two experimental results and confirm the miRNAs associated with steroid hormone synthesis and immune system in *G. rarus*. *fabp3* (fatty acid-binding protein 3, related to apoptosis), *mfap4* (microfibrillar-associated protein 4, immune response with bacterial challenge), *abca1* (ATP-binding cassette transporter A1, cholesterol metabolism), *foxo3* (forkhead transcription factor 3a, TGFβ-induced apoptosis and longevity), *tgfb1* (transforming growth factor β1, disease like apoptosis and tumor), and *zfp36l1* (the tristetraprolin or tristetraprolin family of CCCH tandem zinc finger proteins, TNFα target) were selected for qPCR verification. U6snRNA and *ef1a* were used as endogenous controls (primers presented in Supplementary Table 1) for the selected miRNAs and RNAs, respectively. Amplification efficiencies of the detected genes ranged from 90 and 110%.

### Data Analyses

All data were analyzed by SPSS 19.0 and presented as mean ± SD. One-way ANOVA with Dunn’s *post hoc* test was used. Significance levels or *p*-values were stated in each corresponding figure legend. Significance was accepted at the level of *p* < 0.05 (^∗^*p* < 0.05, ^∗∗^*p* < 0.01). The Levene’s test was used to determine the homogeneity of variance (O’Neill and Mathews, 2002).

## Results

### Morphological Changes

**FIGURE 1:**
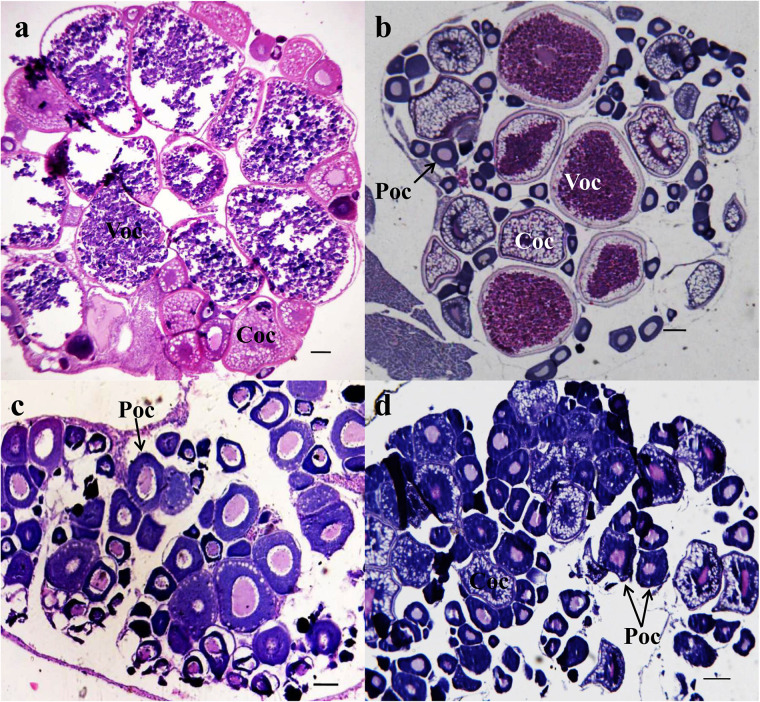
Photomicrographs of transverse ovary sections of adult *Gobiocypris rarus* unexposed and exposed to methyltestosterone (MT) (*n* = 6). H&E staining, scale bars = 150 μm. **(a)** The 7-day control group ovary, **(b)** 25 ng/L of MT for 7-day exposure, **(c)** 50 ng/L of MT for 7-day exposure, and **(d)** 100 ng/L of MT for 7-day exposure. Voc, mature oocyte; Coc, pre-yolk oocyte; Poc, primary oocyte.

The total weight, total length, and body length measured (*n* = 6 in each group) are listed in Table 1. The total weight and body length were significantly decreased in the 50-ng/L MT groups (*p* < 0.01). The major oocytes in the ovaries of the control group were vitellogenic oocytes (Voc) and cortical alveolus stage oocytes (Coc) (Figure 1a). In the 25-ng/L MT exposure groups, some fish had mature ovaries with a relatively lower proportion of mature oocytes (Figure 1b). In the 50-ng/L MT-treated groups, the Coc and perinucleolar oocytes (Poc) were predominant in the ovaries, and the diameter of the Poc was decreased compared with those of the control group (Figures 1c,d).

**TABLE 1:** Biological indicator and the content of hormones in treatment and control groups.

**Group**	**Body length (cm)**	**Total length (cm)**	**Total weight**	**VTG (ng/mg)**	**FSH (mIU/mg)**	**11-KT (pg/mg)**	**E2 (pg/mg)**	**LH (mIU/mg)**	**T (pg/mg**)
Control	3.62 ± 0.14	4.76 ± 0.17	1.26 ± 0.23	233.52 ± 53.45	57.71 ± 0.47	33.54 ± 1.72	4.25 ± 0.14	15.01 ± 0.23	81.00 ± 0.37
25 ng/L methyltestosterone (MT)	3.56 ± 0.55	4.70 ± 0.52	1.20 ± 0.40	181.19 ± 8.69	46.46 ± 0.32*↓	26.44 ± 0.47*↓	3.74 ± 0.41	11.42 ± 1.03*↓	69.67 ± 0.15**↓
50 ng/L MT	3.00 ± 0.21**↓	4.16 ± 0.34	0.77 ± 0.19**↓	531.09 ± 9.51**↑	112.56 ± 1.88**↑	65.96 ± 0.49**↑	9.24 ± 0.19**↑	23.33 ± 1.43**↑	200.03 ± 2.74**↑
100 ng/L MT	3.30 ± 0.33	1.12 ± 0.25	1.12 ± 0.25	231.41 ± 1.51	60.96 ± 4.22	37.06 ± 1.94	5.04 ± 0.20	11.69 ± 1.21*↓	96.37 ± 0.00**↑

*p < 0.01, **p < 0.05.

### Sex Steroid Hormone Activity

The levels of sex steroid hormone activity (*n* = 3) in the female rare minnow in response to MT are shown in Table 1. Gonadal FSH, LH, 11-KT, and T (*p* < 0.01) were significantly decreased after MT exposure at 25 ng/L for 7 days. Gonadal VTH, FSH, LH, 11-KT, E2, and T were significantly increased after exposure to 50 ng/L of MT (*p* < 0.01) for 7 days. Gonadal LH (*p* < 0.05) and T (*p* < 0.01) were significantly decreased and increased, respectively, following MT exposure at 100 ng/L for 7 days.

### RNA- and miRNA-Seq Analysis

**FIGURE 2:**
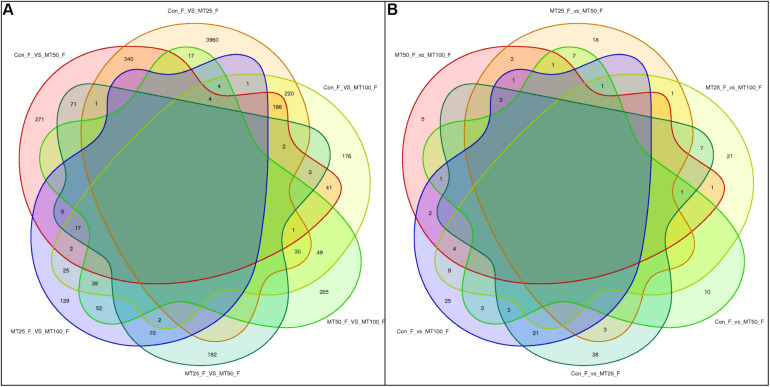
Venn diagram analysis of differentially expressed genes (DEGs). **(A)** DE miRNAs miRNA (DEMs). **(B)** In rare minnow ovaries after MT (25, 50, and 100 ng/L) treatment.

In RNA-seq, the gene number and ratio showed no significant changes across different groups (*n* = 3) (Supplementary Table 2). For miRNA-seq, total non-coding RNA reads, rRNA, snRNA, snoRNA, tRNA, known miRNA num, novel miRNA num, miRNA number, and target gene number showed no significant changes across different groups (*n* = 3). The significant DEGs for normalized gene expression among different MT groups were identified. In comparison with those of the control group, 5,233 (MT25-F vs. Con-F), 1,663 (MT50-F vs. Con-F), and 1,222 (MT100-F vs. Con-F) genes were detected as significant DEGs. The MT50-F and MT100-F groups presented 3,200 and 1,875 significant DEGs, respectively, compared with those of the MT25-F groups, whereas the MT100-F groups presented 801 significant DEGs compared with those of the MT50-F groups (Supplementary Table 3). The results indicated that there were 340 identical DEGs between the MT25-F (Con-F vs. MT25-F) and MT50-F (Con-F vs. MT50-F) groups, 220 identical DEGs between the MT25-F (Con-F vs. MT25-F) and MT100-F (Con-F vs. MT100-F) groups, and 41 identical DEGs between the MT50-F (Con-F vs. MT50-F) and MT100-F (Con-F vs. MT100-F) groups (Figure 2A). For miRNA-seq, 76 (MT25-F), 27 (MT50-F), and 73 (MT100-F) DEMs were identified in comparison with those of the control group (Supplementary Figure 1a). Within the 76, 27, and 73 DEMs in the 25, 50, and 100-ng/L MT group, 17, 8, 24, and 59, 19, 49 were annotated to known and novel miRNA, respectively. There were three identical DEMs in the MT25-F, MT50-F, and MT100-F groups (Figure 2B).

**FIGURE 3:**
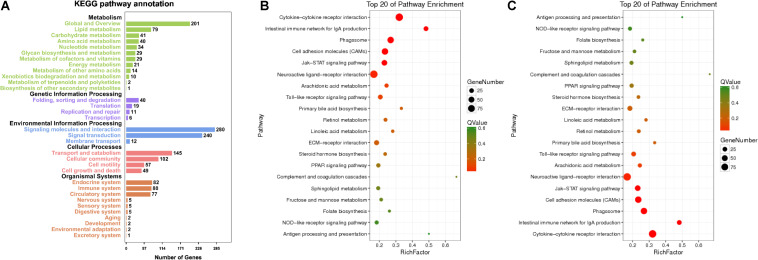
Enriched KEGG pathway annotation of RNA **(A)** and top 20 KEGG pathways of RNA **(B)** and miRNA **(C)**. Statistical summary of distribution of genes in each pathway in each trend. Rich factor refers to the ratio of the number of genes in the pathway entry in the DEGs to the total number of genes in the pathway entry in all genes. The larger the rich factor is, the higher the degree of enrichment is. Q-value is the *p*-value corrected by multiple hypothesis test, and the value range is 0–1. The closer to zero, the more significant the enrichment is. The graph is drawn with the top 20 pathways sorted by Q-value from small to large.

In RNA-seq, several KEGG pathways like cytokine–cytokine receptor interaction, phagosomes, and Jak-STAT signaling pathway were enriched (Figure 3B). The top five KEGG pathways, e.g., metabolism (global and overview, lipid, and carbohydrate metabolism as the top three) were enriched (Figure 3A), while for miRNA-seq, the top five KEGG pathways were similar to those found in RNA-seq (Figure 3C). With respect to RNA-seq, MT concentration explained the largest fraction of the variation (24.8% along PC1, *p* < 0.05; Supplementary Figure 1b) after accounting for the variation present. Approximately 11% of the variation was explained by PC2, while 10.1% of the variation was explained by PC3. In contrast, for miRNA, MT concentration explained the largest fraction of the variation (43.7% along PC1, *p* < 0.05; Supplementary Figure 1b) after accounting for the variation present, 18.7% of the variation was explained by PC2, while 11.9% of the variation was explained by PC3.

### STEM Analysis

**FIGURE 4:**
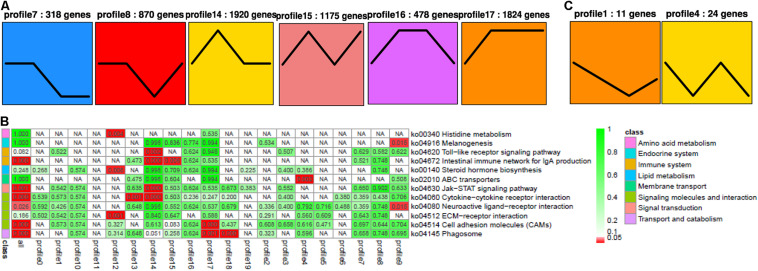
STEM analysis of RNA **(A)** with its relevance **(B)**, and miRNA **(C)**. The ID of the trend and the number of genes in the trend are shown at the top of the figure; the trend block with color (*p* < 0.05): the trend with significant enrichment and the trend block with a similar trend have the same color. In the pathway enrichment analysis *p*-value (Q-value) heat map, the filtering standard is less than or equal to 0.05 in at least one trend. The color bar legend “class” on the left represents the KEGG B class pathway annotation. Each row represents a KEGG pathway annotation, and each column represents a trend or all trends. The number in the grid is the *p*-value/Q-value; “NA” means that the pathway is not enriched in the trend. The darker the lattice color is, the more significant the pathway enrichment is.

For RNA-seq, 318, 870, 1,920, 1175, 478, and 1,824 DEGs were identified in profiles 7, 8, 14, 15, 16, and 17, respectively, by STEM analysis (*p* < 0.05, Figure 4A). All these DEGs were enriched in several pathways (Figure 4C), including Toll-like receptor (except for profile 14), IgA production of intestinal immune network (especially for profiles 14 and 15), Jak-STAT (profile 14), cytokine and its receptor interaction (profile 14), neuroactive ligand–receptor interaction (only in profile 9), cell adhesion molecules (CAMs) (profile 17), and phagosomes (profile 17, Supplementary Table 4). In miRNA-seq, 11 and 24 DEMs were identified in profiles 1 and 4, respectively (*p* < 0.05, Figure 4B and Supplementary Table 5).

**FIGURE 5:**
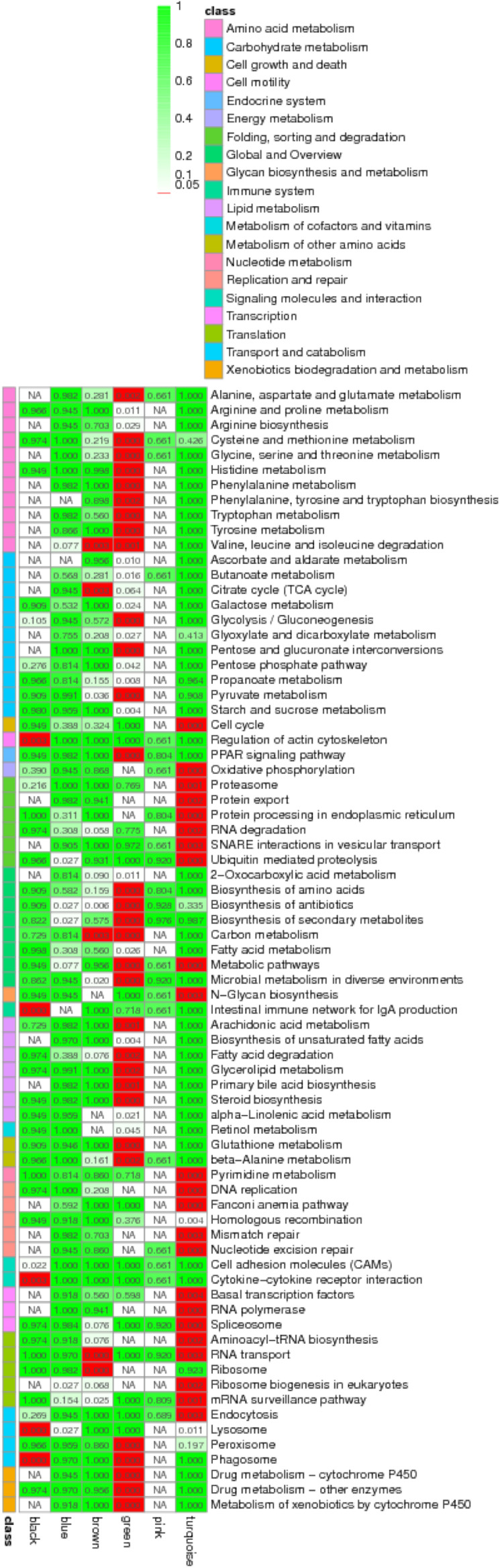
Enriched KEGG pathway annotation for miRNA–mRNA pairs by the weighted gene co-expression network analysis (WGCNA analysis) using Q-value.

Among these small RNAs and RNAs, profile 1 of miRNA was most associated with profiles 14 and 17 of RNA, while it was least associated with profile 16. Furthermore, profile 4 of miRNA was most associated with profiles 8, 14, 15, and 17, while it was least associated with profiles 7 and 16. In general, profiles 14 and 17 were the most related, while 16 was the least related. When we looked for miRNA–target gene interaction network using WGCNA analysis, it showed the highest occurrence in the green and turquoise pathways (Figure 5). Within the green groups, amino acid and carbohydrate metabolism, endocrine system, global and overview, lipid metabolism and metabolism, etc., were enriched, whereas in the turquoise group, cell growth and death, replication and repair, translation, and endocytosis, etc., were enriched.

In addition, a total of 3,949 miRNA–mRNA pairs with negative correlations were identified; moreover, under the state of profile 1, 940 miRNA–mRNA pairs were also different, and 2,663 miRNA–mRNA pairs of profile 4 were identified. Therefore, when miRNAs are induced by MT, their target mRNAs are downregulated and vice versa.

### qPCR Validation of Differentially Expressed Genes

**FIGURE 6:**
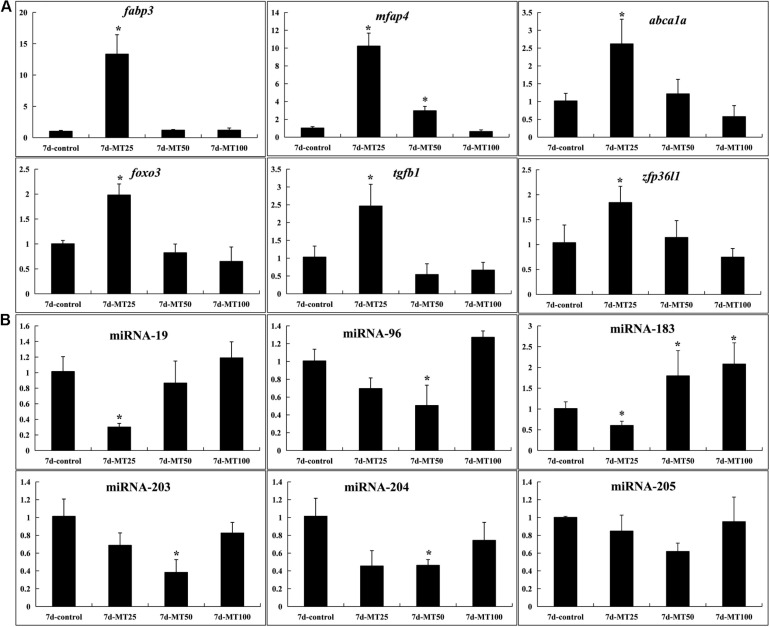
qPCR verification (*n* = 3). *ef1*α and *U6* were used as reference gene for genes and miRNAs, respectively. The data of treatment group and control group were analyzed using the 2^–ΔΔCt^ method. The results were represented as the mean ± SD of female fish. *p < 0.05. **(A)** Ovary RNA. **(B)** Ovary miRNA expression. The name of the genes and their potential functions are as follows: *fabp3* (fatty acid-binding protein 3, related to apoptosis), *mfap4* (microfibrillar-associated protein 4, immune response with bacterial challenge), *abca1* (ATP-binding cassette transporter A1, cholesterol metabolism), *foxo3* (forkhead transcription factor 3a, TGFβ-induced apoptosis and longevity), *tgfb1* (transforming growth factor β1, disease like apoptosis and tumor), and *zfp36l1* (the tristetraprolin or tristetraprolin family of CCCH tandem zinc finger proteins, TNFα target).

Finally, we chose six negative miRNA–mRNA interactions with six mature miRNAs (miR-19, miR-183, miR-203, miR-204, miR-205, and miR-96) and six validated mRNAs (*fabp3*, *mfap4*, *abca1*, *foxo3*, *tgfb1*, and *zfp36l1*). The sequencing results were consistent with the validation with qPCR (*n* = 3). *Abp3*, *mfap4*, *abca1*, *foxo3*, *tgfb1*, and *zfp36l1* were significantly increased in the female rare minnow exposed to 25 ng/L of MT. *Mfap4* was significantly increased in the female fish exposed to 50 ng/L of MT. miRNA-19 and miRNA-183 were significantly decreased in the female rare minnow exposed to 25 ng/L of MT. miRNA-96 and miRNA-203 were significantly decreased in the female rare minnow exposed to 50 ng/L of MT. miRNA-183 was significantly increased in the female fish exposed to 50 ng/L of MT. We also found that several genes played critical roles in multiple pathways. For example, *kirrel* and *eef1a1* showed a negative correlation with miR-430 (Figure 6 and Supplementary Table 5).

## Discussion

A growing number of studies have shown that ovarian development is suppressed by MT exposure (Gao et al., 2014, 2015; Liu S. et al., 2014; Liu Y. et al., 2014; Rutherford et al., 2015; Zheng et al., 2016). In the present study, we found the decreasing Voc numbers in ovaries, indicating that MT could inhibit the gonadal development of female fish when exposed for 7 days. These results suggested that MT may have more disrupting effects on the ovary at an earlier stage, while a possible stress adaptation occurred in fish that weakened these disrupting effects with the prolongation of the exposure period. A previous study indicated that MT inhibited the E2, LH, FSH, LH, 11-KT, and T in *Pseudorasbora parva* (Wang et al., 2020). In the present study, we found that the biological indicators and hormone levels were significantly altered in the MT-treated female fish compared with the other test groups. FSH, LH, 11-KT, and T were significantly decreased in female fish after MT exposure to 25 ng/L for 7 days. 11-KT plays an important role in controlling pre-Voc growth in *A. japonica* (Lai et al., 2018). It indicates that MT inhibits follicle maturation by inhibiting steroid hormones. In Leydig cells of male rat treated with testosterone for 60 days, the transcriptional downregulation of steroidogenic enzymes coupled with significantly decreased LH levels in circulation (Kostic et al., 2011) suggests that MT could regulate androgen production through LH-LHR-cAMP signaling. In the present study, the cause of induced VTG synthesis for MT probably is that MT can be aromatized into methylestradiol (ME2), and ME2 with estrogenic effect subsequently upregulates VTG via the hepatic estrogen receptor (Hornung et al., 2004). The results of our manuscript and previous studies suggest that MT plays roles in the gonadal differentiation and maturation in the rare minnow.

Herein, the involvement of the miRNA–mRNA regulatory network in this phenomenon has not been reported yet. DEGs (924) and DEMs (seven) were identified in this group for mRNA and miRNA, respectively, and these were enriched in metabolic pathways, cytokine–cytokine receptor interaction, etc. The integrated miRNA–mRNA analysis revealed that among those pathways with *p* < 0.05, the latter four pathways were further enriched in the STEM analysis. Six genes (*fabp3*, *mfap4*, *abca1*, *foxo3*, *tgfb1*, and *zfp36l1*) were differentially upregulated in the 25-ng/L MT exposure group. Through RNA sequencing, we successfully identified 924 upregulated and 739 downregulated DEGs in the 50-ng/L MT groups compared with control group, and these were concentrated in profiles 14 and 17. Moreover, from miRNA sequencing, we successfully identified seven upregulated and 20 downregulated miRNAs, which were identified in profiles 1 and 4. Among these, miR-19, miR-183, miR-203, miR-204, miR-205, and miR-96 were downregulated. It has been reported that retinoic acid and *cyp26a1* are necessary only during the early stages of somatogenesis (Sirbu and Duester, 2006), wherein it represses the expression of miR-19 family members as the 3′UTR of *cyp26a1* as a *bona fide* target of miR-19, which was identified using *in vivo* reporter analysis (Franzosa et al., 2013). The present study showed that the targeted genes of miR-19, *fabp3* and *tgfb1*, were upregulated following MT exposure.

In another study, miR-19 was reported to directly target the TGFβ pathway associated with the inflammation pathway (Li et al., 2012), whereas the miR-130a–*fabp3* pair was reported to play a vital role in the PI3K/AKT–mTOR pathway (Chen et al., 2018). Recent studies reported that miR-96 was important in otic vesicle development, involved in the hearing process, along with miR-183 (target *mfap4*) (Li et al., 2010; Kim et al., 2018), and in the formation of the nervous system in conjunction with miR-184 (Li et al., 2016, 2019). LncRNA UCA1 promotes cell proliferation by upregulating Foxo3 and downregulating miR-96 (Zhou et al., 2018). Furthermore, miR-96, along with miR-200 (target *kirrel*; *kirrel1*) mutations, causes steroid-resistant nephrotic syndrome (Solanki et al., 2019) and is essential for steroid synthesis during early sex differentiation in tilapia (Tao et al., 2016). *mfap4* is also shown to play an important role in the innate immune system of zebrafish (Zakrzewska et al., 2010; Li et al., 2014; Walton et al., 2015) and catfish (Niu et al., 2011). Foxo3a and Foxo3b in ovarian follicular cells during vitellogenesis were significantly increased stage dependently and co-localized with CYP19a1a (Liu et al., 2016). *foxo3b* retained most of the functions including upregulating *cyp19a1a* during vitellogenesis of orange-spotted grouper (Liu et al., 2016). WB revealed that overexpression of miRNA-96 substantially reduced FOXO3 protein expression (Yin et al., 2020). In the present study, qRT-PCR and miRNA-seq indicated that miRNA-96 decreased, while *foxo3* increased, in ovaries of rare minnow after MT exposure. The miR-183 cluster (miR-96/183)–*foxo3* pair presented in this study revealed that its pathway may result in damage to the central nervous system (Li et al., 2010), immune impairment, or even cancer (Dambal et al., 2015; Ichiyama and Dong, 2019; Zou et al., 2019). The latest study showed that miR-101 regulated *STAR*, *CYP19A1*, *CYP11A1*, and *3*β*-HSD* steroid hormone synthesis-associated genes by *STC1* depletion, thus, promoting E2 secretions (An et al., 2020). These results suggest that MT regulates related gene expression by interfering with miRNAs, thereby, injuring the ovary.

The members of miR-203, miR-204, and miR-205 targeted the *mfap4* gene, which is involved in immune response (Kang et al., 2019) through miR-203–Irak4–Nf-κB-mediated signaling (Xu et al., 2018). Zebrafish miR-203 targeted *dmrt2b* associated with muscle differentiation (Lu et al., 2017), *pax6b* related to retina development (Rajaram et al., 2014), as well as the Wnt signaling transcription factor *lef1* essential for caudal fin regeneration (Thatcher et al., 2008). Moreover, miR-183 cluster, together with miR-203 (target *mfap4*), is expressed in normal T cells involved in C/EBPβ pathway (Steinhilber et al., 2015) through the suppressor of cytokine signaling-3 (Socs-3, Sonkoly et al., 2007). Our results suggest that miR-203 may, thus, be involved in the process of immune response, organic differentiation, and development. *Cyp19a1a* directly participates in the regulation of sexual reproduction in teleost fish (Liu S. et al., 2014; Liu Y. et al., 2014). Estradiol-17β (E2) is produced by conversion of androgen via cytochrome P450 aromatase, encoded by *cyp19a1a*. Thus, the expression of *cyp19a1a* and E2 secretion plays important roles in sex differentiation, gonadal development, and sex reversal. The transcriptional modulation of steroidogenic enzymes in response to MT could be triggered by factors in the HPG axis.

## Conclusion

In general, in this study, 73,449 unigenes, 24 known mature miRNAs, and 897 novel miRNAs of *G. rarus* were identified by integrated analysis of mRNA- and miRNA-seq. Among them, we successfully identified six miRNA–target pairs, which suggests that they might possibly be involved in cell proliferation and development, signal transduction, metabolic and immune processes, and in the development and functioning of the nervous system. *fabp3 and tgfb1* are target genes regulated by miR-19, while *abca1*, *foxo3*, *tgfb1*, and *zfp36l1* are target genes regulated by miR-96, and *mfap4* and *foxo3* are target genes regulated by miR-183, while *mfap4* is also regulated by miR-203. Such mRNAs and miRNAs, corresponding with ovarian development and innate immune response, were tested by qPCR. The differentially expressed miRNAs (miR-183 cluster and miR-203) with MT administration are provided as the novel regulators in the process of ovarian development and innate immune system in *G. rarus*. Altogether, these results will aid to improve the current understanding of the toxicological effects on fish in response to androgen and will lay a foundation for further studies of EDCs.

## Data Availability Statement

The datasets presented in this study can be found in online repositories. The names of the repository/repositories and accession number(s) can be found below: NCBI, PRJNA730106.

## Ethics Statement

The animal study was reviewed and approved by the Institutional Animal Care and Use Committee of Shanxi Agriculture University. Written informed consent was obtained from the owners for the participation of their animals in this study.

## Author Contributions

SL designed the experiment and wrote the manuscript. QY and YC conducted the qRT-PCR and histological experiment. QL, WW, and JS contributed to sequencing data analysis. All authors contributed to the article and approved the submitted version.

## Conflict of Interest

The authors declare that the research was conducted in the absence of any commercial or financial relationships that could be construed as a potential conflict of interest.

## Publisher’s Note

All claims expressed in this article are solely those of the authors and do not necessarily represent those of their affiliated organizations, or those of the publisher, the editors and the reviewers. Any product that may be evaluated in this article, or claim that may be made by its manufacturer, is not guaranteed or endorsed by the publisher.

## Supplementary Material

Supplementary Figure 1 PCA analysis of RNA and miRNA. The variation between samples was constrained in the PCA analysis [**(a)** 24.8 and **(b)** 43.7% of the overall variance for RNA and miRNA; *P* < 0.05]. In both panels, different colors correspond to samples from different MT concentrations. The percentage of variation explained by each axis refers to the proportion of the total data variance explained by the constrained factor.

Supplementary Figure 2 Heatmaps of RNA **(a)** and miRNA **(b)** (*n* = 3).

Supplementary Figure 3 Network heatmap of RNA and miRNA. **(a)** Analyzing the correlation between the two modules and drawing a heat map. Each row and column represents a module, the number in the box is the correlation coefficient of the two modules, and the number in brackets is the *p*-value. The darker the square color is (red or green), the stronger the correlation is; the lighter the square color, the weaker the correlation. The *p*-value of the correlation between the two modules is calculated by student’s *t*-test. The smaller the p-value, the higher the similarity between the two modules. **(b)** Heat map drawn from clusters of the module genes. Each row and column represent a gene, and the darker the color of each point (white→yellow→red) represents the stronger connectivity between the two genes corresponding to the row and column. *P*-value was calculated by student’s *t*-test. The smaller the *p*-value, the more significant the correlation between the gene and the module. **(c)** The value of module eigenvalue in each sample reflects the comprehensive expression level of all genes in each sample. The abscissa is the sample, the ordinate is the module, and the characteristic value of the module is used for drawing. Red represents high expression and green represents low expression. The graph directly reflects the expression mode of each module in each sample.
