# Supplementary material for: Integrated Analysis of mRNA- and miRNA-Seq in the Ovary of Rare Minnow Gobiocypris rarus in Response to 17α-Methyltestosterone
Source: Front Genet. 2021 Aug 5;12:695699. doi: 10.3389/fgene.2021.695699 (PMC8375321; doi:10.3389/fgene.2021.695699)
Supplement: Supplementary Figure 1 — PCA analysis of RNA and miRNA. The variation between samples was constrained in the PCA analysis [(a) 24.8 and (b) 43.7% of the overall variance for RNA and miRNA; P < 0.05]. In both panels, different colors correspond to samples from different MT concentrations. The percentage of variation explained by each axis refers to the proportion of the total data variance explained by the constrained factor. [file Presentation_1.pdf]

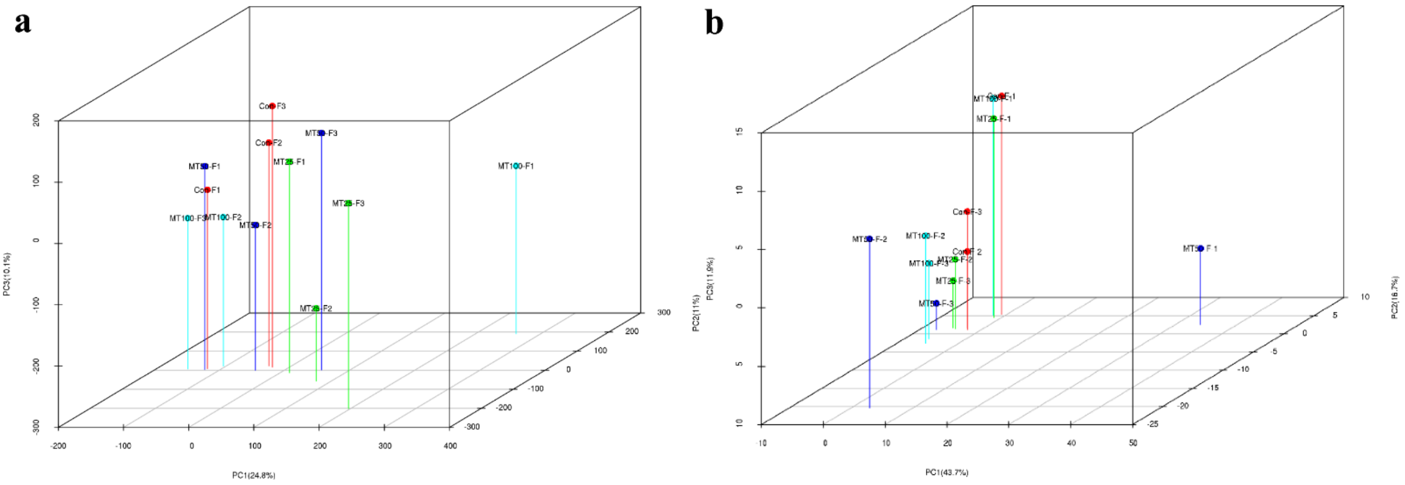

Fig S1 PCA analysis of RNA and miRNA. The variation between samples was constrained in the PCA analysis (24.8% and 43.7% of the overall variance for RNA and miRNA; $P < 0.05$). In both panels, different colors correspond to samples from different MT concentrations. The percentage of variation explained by each axis refers to the proportion of the total data variance explained by the constrained factor.

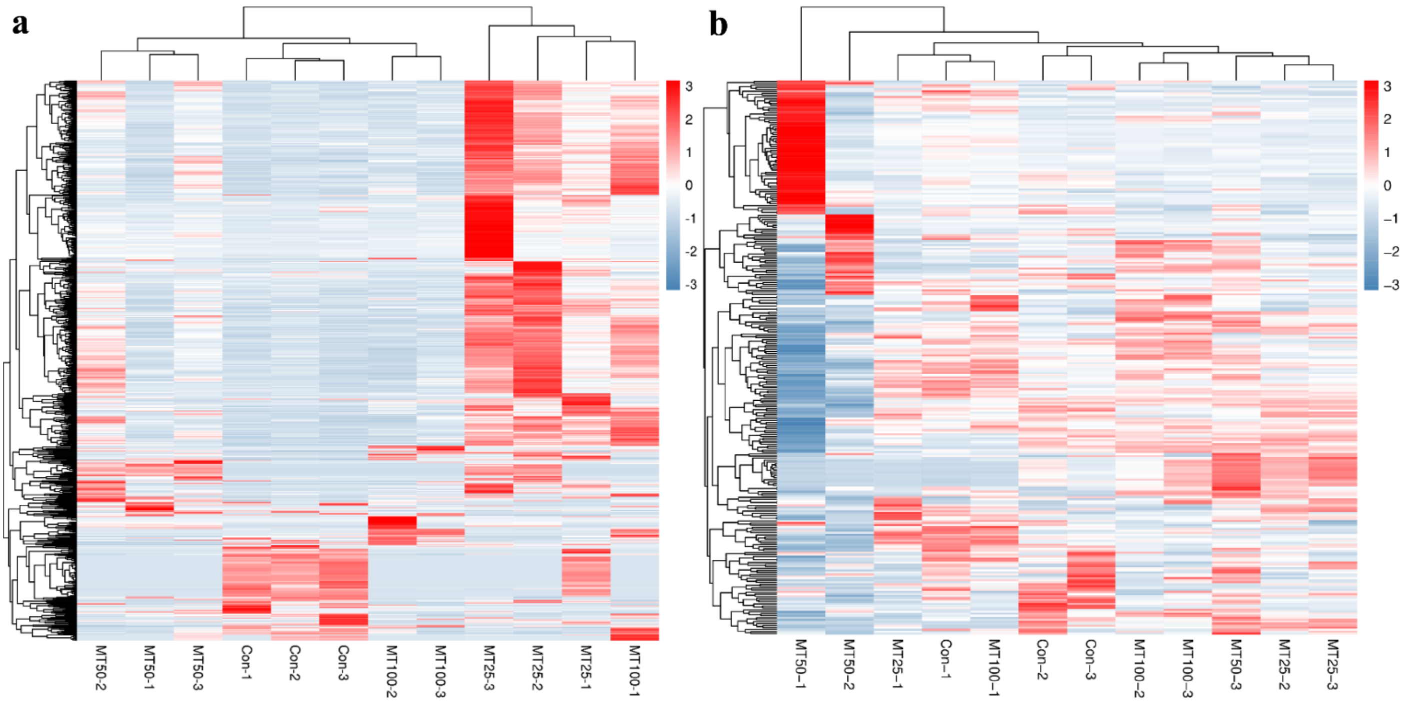

Fig S2 Heatmaps of RNA (a) and miRNA (b) (*n*=3).

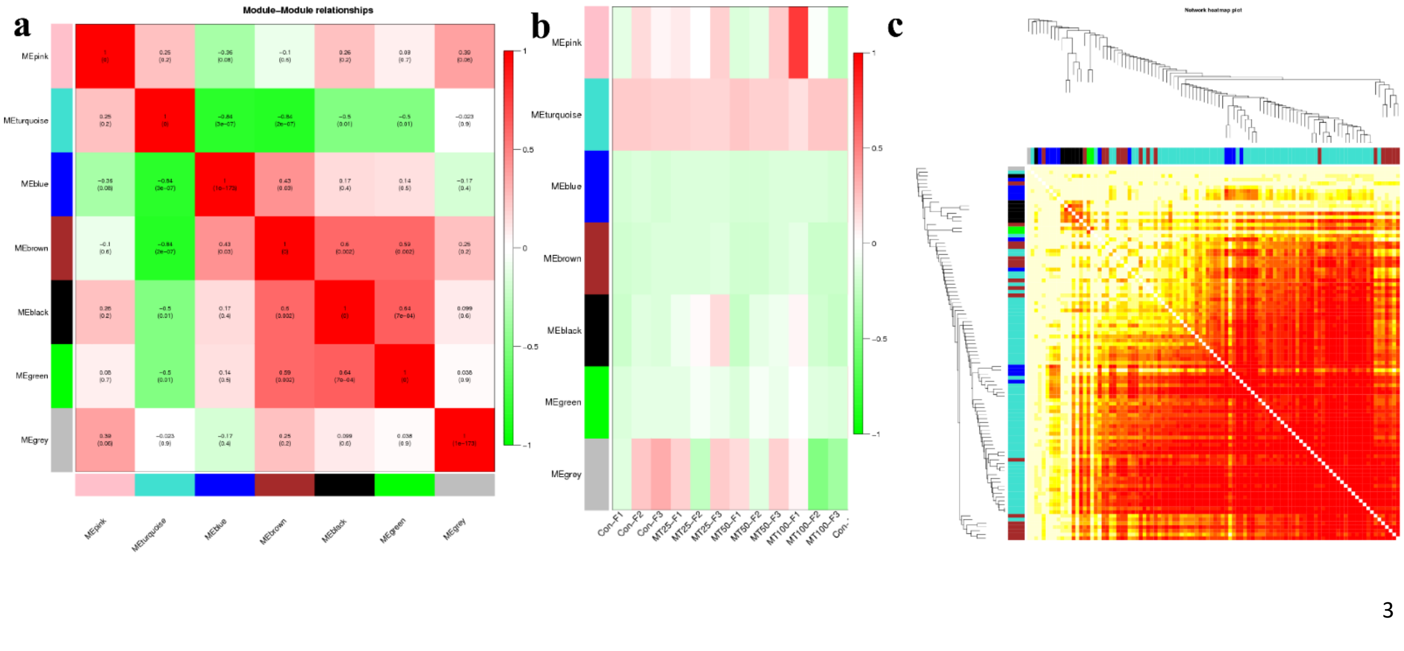
a
Module–Module relationships
MEpink
MEturquoise
MEblue
MEbrown
MEblack
MEgreen
MEgrey
b
Con–F1
Con–F2
Con–F3
MT25–F1
MT25–F2
MT25–F3
MT50–F1
MT50–F2
MT50–F3
MT100–F1
MT100–F2
MT100–F3
c
Network heatmap plot

Fig S3 Network heatmap of RNA and miRNA. (a) analyzing the correlation between the two modules and drawing a heat map. Each row and column represents a module, the number in the box is the correlation coefficient of the two modules, and the number in brackets is the p-value. The darker the square color is (red or green), the stronger the correlation; the lighter the square color, the weaker the correlation. The p-value of the correlation between the two modules is calculated by student's t-test. The smaller the p-value, the higher the similarity between the two modules. (b) heat map drawn from clusters of the module genes. Each row and column represents a gene, and the darker the color of each point (white→yellow→red) represents the stronger connectivity between the two genes corresponding to the row and column. P-value was calculated by student's t-test. The smaller the p-value, the more significant the correlation between the gene and module. (c) the value of module eigenvalue in each sample reflects the comprehensive expression level of all genes in each sample. The abscissa is the sample, the ordinate is the module, and the characteristic value of the module is used for drawing. Red represents high expression and green represents low expression. The graph directly reflects the expression mode of each module in each sample.

Table S1 Primer sequences for qPCR. The original sequence was acquired from the *G. rarus* transcriptome database compiled in the present study. Primers were designed based on the original sequence.

**Gene**	**primer 5'-3'**

fabp3	F:TTGAACTGAGCCTGGCATCT<br>R:CGGACCAAACCCAACCTG
mfap4	F:GGGTTGTGAAACTGATGGGT<br>R:TGGTCTTTGTCAAGGGTGGT
abca1	F:CAACAACCCTTGCTTTCGC<br>R:CAACAACCCTTGCTTTCGC
foxo3	F:CAACAACCCTTGCTTTCGC<br>R:CCAAAACTGAAAGTAGAAGACCCT
tgfb1	F:GGAACTGTATCGTGGAGTAGGTG<br>R:CCAACATCTCGTTTCCTGCG
zfp36l1	F:GATTTCCTCCCCTGGCAAC<br>R:TCCCCTCGTGGATGAAGTGA
ef1a	F:ACAAATGCGGTGGAATCG<br>R:TCAAACTTCCAGAGAGCGATA
u6	GCGCGGCCTGCATTGTACACAC
mir-19	AGCTTTGCGGGGTGGGCAGT
mir-96	CGGCCTTTGGCACTAGCACATTTTTGCT
mir-183	GCCGGCTATGGCACTGGTAGAATTCACT
mir-203	GCCGCAGTGGTTCTCAACAGTTCAACA
mir-204	GCCGGTTCCCTTTGTCATCCTATGCCT
mir-205	GCCCTCCTTCATTCCACCGGAGTCTG

Table S2 Quality of sequencing data (n=3).

Sample	Genes Num-RNA	Ratio-RNA	total	rRNA	snRNA	snoRNA	tRNA	known mirna num	novel mirna num	miRNA_number	target_gene_number
Con	46738±2835	0.64±0.04	905412±190784	10491±3933	790±303	257±4	2525±686	394±66	476±67	871±46	23528±86
MT25-F	52206±1809	0.71±0.02	835339±14155	14381±4534	697±190	302±10	2816±605	413±32	476±84	889±57	23586±88
MT50-F	47544±3812	0.65±0.05	901563±144465	10225±5877	521±271	227±116	2218±678	364±38	439±123	803±103	23410±162
MT100-F	47364±5283	0.64±0.07	720898±170967	11826±3437	607±65	285±34	2133±220	414±28	471±113	886±93	23561±152

Table S3 Statistical analysis of DEGs and DEMs (*n*=3). In the comparison of 25 ng L^{-1} 17MT group with the controls, 5233 DEGs (including 4746 upregulated and 487 downregulated genes) were identified. In the comparison of 25 ng L^{-1} 17MT group with the controls, 76 of the total GO clusters (including 25 upregulated and 51 downregulated genes) were identified. Table description: (1) Pair: control verus experimental group name; (2) Up: the number of upregulated genes with significant differences; (3) Down: the number of downregulated genes with significant diffevel: novel miRNAs.

**Pair**	**DGEs, DEMs/known/novel (Up)**	**DGEs, DEMs/known/novel (Down)**
Con-F-VS-MT25-F	4746, 25/7/18	487, 51/10/41
Con-F-VS-MT50-F	924, 7/3/4	739, 20/5/15
Con-F-VS-MT100-F	781, 31/13/18	441, 42/11/31
MT25-F-VS-MT50-F	353, 17/11/6	2847, 21/6/15
MT25-F-VS-MT100-F	349, 28/9/19	1526, 17/6/11
MT50-F-VS-MT100-F	480, 16/6/10	321, 6/2/4

Table S4 The significant top 5 KEGG pathways gathered according to the comparison between different concentrations of 17MT addition. 114 hits have been matched in metabolic KEGG pathways in 25 ng/L 17MT groups when compared with controls.

Comparison	Top 5 pathways	hits	Qvalue
Con-F-VS-MT25-F	Metabolic pathways	114	1.00E+00
	Cytokine-cytokine receptor interaction	64	1.16E-12
	Neuroactive ligand-receptor interaction	52	7.01E-01
	Cell adhesion molecules (CAMs)	46	2.09E-05
	Phagosome	43	8.74E-06
Con-F-VS-MT50-F	Metabolic pathways	51	7.62E-01
	Phagosome	28	3.61E-09
	Neuroactive ligand-receptor interaction	23	3.12E-01
	Focal adhesion	18	4.20E-01
	Calcium signaling pathway	18	7.62E-01
Con-F-VS-MT100-F	Metabolic pathways	39	0.407324
	Neuroactive ligand-receptor interaction	16	0.407324
	Cell adhesion molecules (CAMs)	13	0.17807
	MAPK signaling pathway	12	0.793473
	Cardiac muscle contraction	10	0.231281
MT25-F-VS-MT50-F	Metabolic pathways	66	1.00E+00
	Cytokine-cytokine receptor interaction	44	1.61E-10
	Endocytosis	30	3.00E-01
	Phagosome	28	2.12E-04
	Neuroactive ligand-receptor interaction	28	1.00E+00
MT25-F-VS-MT100-F	Metabolic pathways	59	8.77E-01
	Cytokine-cytokine receptor interaction	32	6.44E-08
	Neuroactive ligand-receptor interaction	31	6.33E-02

	Phagosome	17	6.33E-02
	Calcium signaling pathway	17	9.97E-01
MT50-F-VS-MT100-F	Metabolic pathways	24	0.811569
	Cardiac muscle contraction	11	0.005672
	Neuroactive ligand-receptor interaction	11	0.568864
	Cell adhesion molecules (CAMs)	10	0.085882
	Oxidative phosphorylation	9	0.014875

Table S5 The mRNA and miRNA profile in the comparison by STEM analysis.

**Profile ID**	**Con-F**	**MT25-F**	**MT50-F**	**MT100-F**
pro1				
miR-146-x	179545	122963	25050	55766
miR-451-y	6.88	3.30	1.76	4.40
miR-8796-x	15.42	2.13	0.51	3.53
novel-m0170-5p	202.26	110.66	75.69	91.31
novel-m0617-5p	68.23	30.21	16.08	27.42
novel-m0776-5p	6.24	2.70	1.76	2.92
novel-m0271-5p	9.76	4.52	0.49	2.61
novel-m0753-5p	9.76	4.52	0.49	2.61
novel-m0372-5p	5.86	2.64	1.01	1.81
novel-m0362-5p	2.91	0.36	0.01	0.92
novel-m0296-5p	2.57	0.94	0.01	0.22
ID-pro4				
miR-1290-y	11.64	0.01	3.53	0.01
miR-484-z	20.60	0.64	7.61	2.57
miR-147-x	1.76	0.01	1.89	0.15
miR-136-y	1.59	0.15	6.82	0.52
miR-335-y	3.90	0.01	0.98	0.01
miR-185-x	2.47	0.01	1.33	0.01
novel-m0031-5p	8.69	1.41	35.22	1.44
novel-m0443-5p	13.55	3.92	21.97	4.41

novel-m0764-5p	24.23	12.69	20.31	8.17
novel-m0859-3p	34.92	15.44	62.18	11.26
novel-m0875-3p	7.20	2.03	17.46	4.77
novel-m0832-3p	19.58	5.51	30.41	9.67
novel-m0021-3p	19.58	5.51	30.41	9.67
novel-m0210-5p	10.84	4.38	11.24	6.75
novel-m0160-5p	13.00	4.85	29.50	6.88
novel-m0225-3p	22.26	12.78	45.61	9.89
novel-m0130-3p	4.66	2.24	8.63	1.47
novel-m0797-5p	6.10	2.38	5.30	2.70
novel-m0273-5p	9.77	4.63	9.92	4.65
novel-m0883-5p	8.89	3.79	5.84	1.98
novel-m0205-5p	12.13	4.26	9.74	4.49
novel-m0705-3p	4.04	0.36	7.31	0.92
novel-m0627-5p	4.81	0.43	20.96	2.20
novel-m0175-5p	1.84	0.01	1.01	0.01
miR-19	7.09	2.88	3.37	4.46
miR-96	15.42	2.13	0.51	3.53
miR-183	522.76	347.75	1285.02	755.92
miR-203	327.23	647.63	583.06	976.38
miR-204	0.01	0.60	0.49	1.38
miR-205	30.52	34.25	141.11	68.12
abca1	6.63	14.02	8.50	10.80
fabp3	109.03	558.06	289.40	171.05
foxo3	1.71	5.62	3.06	2.84
mfap4	1.06	23.51	28.71	23.08
tgfb1	0.87	2.46	1.05	1.17
zfp36l1	8.44	29.97	11.89	13.42
